# MYBL2 Drives Prostate Cancer Plasticity: Inhibiting Its Transcriptional Target CDK2 for RB1-Deficient Neuroendocrine Prostate Cancer

**DOI:** 10.1158/2767-9764.CRC-24-0069

**Published:** 2024-09-02

**Authors:** Beatriz German, Sarah A. Alaiwi, Kun-Lin Ho, Jagpreet S. Nanda, Marcos A. Fonseca, Deborah L. Burkhart, Anjali V. Sheahan, Hannah E. Bergom, Katherine L. Morel, Himisha Beltran, Justin H. Hwang, Matthew L. Freedman, Kate Lawrenson, Leigh Ellis

**Affiliations:** 1 Department of Surgery, Center for Prostate Disease Research, Murtha Cancer Center Research Program, Uniformed Services University of the Health Sciences, Bethesda, Maryland.; 2 Walter Reed National Military Medical Center, Bethesda, Maryland.; 3 The Henry M. Jackson Foundation for the Advancement of Military Medicine, Inc., Bethesda, Maryland.; 4 Genitourinary Malignancies Branch, Center for Cancer Research, National Cancer Institute, Bethesda, Maryland.; 5 Section of Cardiovascular Medicine, Yale University School of Medicine, New Haven, Connecticut.; 6 Department of Urology, Cedars-Sinai Medical Center, Samuel Oschin Comprehensive Cancer Institute, Los Angeles, California.; 7 Department of Obstetrics and Gynecology and the Women’s Cancer Program at the Samuel Oschin Comprehensive Cancer Institute, Cedars-Sinai Medical Center, Los Angeles, California.; 8 Department of Cancer Biology, Dana-Farber Cancer Institute, Harvard Medical School, Boston, Massachusetts.; 9 Department of Oncologic Pathology, Dana-Farber Cancer Institute, Harvard Medical School, Boston, Massachusetts.; 10 Department of Medicine, University of Minnesota-Twin Cities, Minneapolis, Minnesota.; 11 South Australian Immunogenomics Cancer Institute, University of Adelaide, Adelaide, Australia.; 12 Department of Medical Oncology, Dana-Farber Cancer Institute, Harvard Medical School, Boston, Massachusetts.; 13 Center for Bioinformatics and Functional Genomics, Samuel Oschin Comprehensive Cancer Institute, Cedars-Sinai Medical Center, Los Angeles, California.

## Abstract

Phenotypic plasticity is a recognized mechanism driving therapeutic resistance in patients with prostate cancer. Although underlying molecular causations driving phenotypic plasticity have been identified, therapeutic success is yet to be achieved. To identify putative master regulator transcription factors (MR-TF) driving phenotypic plasticity in prostate cancer, this work utilized a multiomic approach using genetically engineered mouse models of prostate cancer combined with patient data to identify MYB proto-oncogene like 2 (MYBL2) as a significantly enriched transcription factor in prostate cancer exhibiting phenotypic plasticity. Genetic inhibition of *Mybl2* using independent murine prostate cancer cell lines representing phenotypic plasticity demonstrated *Mybl2* loss significantly decreased *in vivo* growth as well as cell fitness and repressed gene expression signatures involved in pluripotency and stemness. Because MYBL2 is currently not druggable, a MYBL2 gene signature was employed to identify cyclin-dependent kinase-2 (CDK2) as a potential therapeutic target. CDK2 inhibition phenocopied genetic loss of *Mybl2* and significantly decreased *in vivo* tumor growth associated with enrichment of DNA damage. Together, this work demonstrates MYBL2 as an important MR-TF driving phenotypic plasticity in prostate cancer. Furthermore, high MYBL2 activity identifies prostate cancer that would be responsive to CDK2 inhibition.

**Significance:**

Prostate cancers that escape therapy targeting the androgen receptor signaling pathways via phenotypic plasticity are currently untreatable. Our study identifies MYBL2 as a MR-TF in phenotypic plastic prostate cancer and implicates CDK2 inhibition as a novel therapeutic target for this most lethal subtype of prostate cancer.

## Introduction

Second-generation androgen deprivation therapies have provided significant life-extending therapies for patients with recurrent or metastatic castration-resistant prostate cancer (mCRPC). Most recently, a subset of these mCRPC tumors progress after androgen deprivation therapies via phenotypic plasticity that allows the cell to adopt phenotypes that no longer rely on androgen receptor (AR) expression and signaling (CRPC-AI). Genetic aberrations such as amplification of MYCN and AURKA (1) and concurrent loss-of-function mutations or copy-number loss events in PTEN, TP53, and RB1 tumor suppressor genes (2) are associated with CRPC-AI. These tumors exhibit multilineage cell identity that includes neuroendocrine (NE) features, a stem or basal cell–like phenotype, altered kinase signaling, altered master regulator transcription factor (MR-TF) expression, and epigenetic alterations. Currently, there are no therapeutic options to provide these patients with durable response, and it is crucial that molecular mechanisms representing actionable therapy targets are identified.

MYB proto-oncogene like 2 (MYBL2) is one of the three members of the MYB transcription factor (TF) family along with MYB and MYBL1 (3). The principal functions of MYBL2 are the regulation of cell proliferation (4), cell survival and apoptosis (5), cell-cycle progression, and the control of cyclin-dependent kinase (CDK) expression and activity which makes MYBL2 an essential component of the DREAM multiprotein complex (dimerization partner, RB-like proteins, E2F2, and MuvB core; refs. 6–8). The DREAM complex is frequently affected in cancer, and the overexpression of MYBL2 has been observed in various aggressive subtypes of tumors and associated with poor clinical prognosis (9–13). MYBL2 is highly regulated at both transcriptional (E2F1, E2F2, and E2F3) and posttranscriptional (CDKs; ref. 14) levels as well as by their catalytic partners including cyclin A and E–CDK2 complex which is required during the S phase. Outside of its more canonical functions, MYBL2 expression is found to be significantly enriched in stem cells or cells undergoing reprogramming when compared with somatic cells (15, 16), implicating a role for MYBL2 as a pluripotency gene. More recently, it was shown that MYBL2 is a chromatin-bound partner for known pluripotency factors OCT4, NANOG, and SOX2 (17). In addition, knockout (KO) of *Mybl2* in mice failed to develop past the blastocyst stage which phenocopied mice exhibiting *Sox2* or *Oct4* KO. The MYBL2 expression level has further been shown to be critical for reprogramming via chromatin remodeling which alters the accessibility of important TFs driving the reprogramming process (18). These studies collectively show that MYBL2 forms an important part of a pluripotency network.

Specific to prostate cancer, gene expression analysis from mCRPC human samples and xenografts revealed high expression of MYBL2 (4, 19–21). An independent study confirmed a significant increase of MYBL2 in CRPC samples, and it was demonstrated that high MYBL2 could facilitate castration resistance by promoting YAP1 activity (22). Recently, increased gene expression of MYBL2 in hormone-sensitive prostate cancer was associated with worse clinical outcomes (19). Furthermore, knockdown of MYBL2 significantly inhibited the proliferation of prostate cancer cell lines in the absence of androgens (20). These studies do provide evidence for a role of MYBL2 driving resistance to AR signaling inhibition.

In the present study, using genetically engineered mouse models (GEMM) and human gene expression data, we demonstrate the enrichment of MYBL2 expression and function in prostate cancers displaying phenotypic plasticity when compared with their adenocarcinoma counterparts. Moreover, loss of *Mybl2* decreased signaling pathways related to pluripotency and proliferation highlighting the important role that MYBL2 plays in orchestrating prostate cancer cell stemness. MYBL2 is currently not druggable, so using a MYBL2 gene signature, we identified CDK2 as a potential therapeutic target for phenotypic plastic prostate cancer. CDK2 inhibition phenocopied genetic loss of Mybl2 and significantly reduced tumor growth. Together, these data indicate that CDK2 inhibition represents a novel therapeutic approach to treat prostate cancers progressing on AR signaling inhibition and exhibiting phenotypic plasticity that includes increased expression and function of MYBL2.

## Materials and Methods

### Animal ethics statement

All experiments involving mice were approved by and performed in accordance with the guidelines of the Cedars-Sinai Medical Center Institutional Animal Care and Use Committee (Animal protocol #9828).

### Cell culture, reagents, and drug response assays

Murine PBCre4:Ptenf/f:Rb1f/f KO (DKO) two-dimensional (2D) cell lines were generated from previously described GEMMs (23). PBCre4:Ptenf/f:Trp53f/f KO (PPKO) 2D cell lines were kindly provided by Dr. Zongbing You (Department of Structural and Cellular Biology, Tulane University School of Medicine, New Orleans, LA; ref. 24). DKO and PPKO cell lines were cultured in high-glucose DMEM and 10% FBS. For drug sensitivity assays, 500 or 350 cells/well for DKO or PPKO, respectively, were seeded into a 96-well plate and treated with the indicated concentrations of BGG463 (CDK2i) and CPS2 (CDK2 degrader) for 72 hours. Cell growth was assessed using the CellTiter-Glo (Promega) assay and crystal violet assay according to manufacturer’s instructions. Cell viability/death was determined by Click-iT Plus EdU and propidium iodide (PI) staining (Thermo Fisher Scientific).

### Assay for Transposase-Accessible Chromatin using sequencing

Assay for Transposase-Accessible Chromatin using sequencing (ATAC-seq) experiment was performed as described (25). In brief, cells were collected by incubating in trypsin for 5 minutes at room temperature and subsequent centrifugation at 592 *g* for 5 minutes at 4°C. Fifty thousand cells were used for tagmentation by incubating in 50 μL of 1× THS-seq buffer [25 μL 2× THS buffer (66 mmol/L Tris acetate, pH 7.8, 132 mmol/L potassium acetate, 20 mmol/L magnesium acetate, and 32% dimethylformamide), 5 μL 10× digitonin, and 2 μL Illumina-TDE1] for 20 minutes at 37°C. To stop the tagmentation reaction, an equal volume of 2× Tagmentation Stop Buffer [10 mmol/L Tris-HCl (pH 8.0) and 20 mmol/L EDTA (pH 8.0)] was added to the reaction and incubated for 10 minutes on ice. For cell lysis, an equal volume of 2× lysis buffer [100 mmol/L Tris-HCl (pH 8.0), 100 mmol/L NaCl, 40 μg mL^−1^ proteinase K, and 0.4% SDS] was added to the tagmentation mix and incubated at 65°C for 15 minutes. The tagmented DNA library was purified in 20 μL buffer EB using the QIAquick PCR Purification Kit (Qiagen). The number of amplification cycles was determined, and library quantification was performed. Paired-end sequencing was performed on Illumina NextSeq 500.

### Chromatin immunoprecipitation sequencing analysis

The chromatin immunoprecipitation sequencing (ChIP-seq) experiment was performed as previously described (23). In brief, fresh-frozen prostatic tumor tissue was pulverized (cryoPREP Pulvrizer, Covaris), resuspended in PBS + 1% formaldehyde, and incubated at room temperature for 20 minutes. Fixation was stopped by the addition of 0.125 mol/L glycine (final concentration) for 15 minutes at room temperature, followed by washing in ice cold PBS + EDTA-free protease inhibitor cocktail (#04693132001, Roche). Chromatin was isolated from biological triplicates by the addition of lysis buffer [0.1% SDS, 1% Triton X-100, 10 mmol/L Tris-HCl (pH 7.4), 1 mmol/L EDTA (pH 8.0), 0.1% NaDOC, 0.13 mol/L NaCl, and 1× protease inhibitor cocktail] + sonication buffer (0.25% sarkosyl and 1 mmol/L DTT) to the samples, which were maintained on ice for 30 minutes. Lysates were sonicated (E210 Focused-ultrasonicator, Covaris), and the DNA was sheared to an average length of ∼200 to 500 bp. Genomic DNA (input) was isolated by treating sheared chromatin samples with RNase (30 minutes at 37°C), proteinase K (30 minutes at 55°C), and de-crosslinking buffer [1% SDS, 100 mmol/L NaHCO3 (final concentration), 6–16 hours at 65°C], followed by purification (#28008, Qiagen). DNA was quantified on a NanoDrop spectrophotometer, using the Quant-iT High-Sensitivity dsDNA Assay Kit (#Q33120, Thermo Fisher Scientific). On ice, H3K27ac (5 μL, catalog no. C15410196; Diagenode Diagnostics) antibody was conjugated to a mix of washed Dynabeads Protein A and Protein G (Thermo Fisher Scientific) and incubated on a rotator (overnight at 4°C) with 1.3 μg of chromatin. ChIPed complexes were washed, sequentially treated with RNase (30 minutes at 37°C), proteinase K (30 minutes at 55°C), de-crosslinking buffer [1% SDS and 100 mmol/L NaHCO3 (final concentration), 6–16 hours at 65°C], and purified (#28008, Qiagen). The concentration and size distribution of the immunoprecipitated DNA were measured using the Bioanalyzer High-Sensitivity DNA kit (#5067-4626, Agilent). Dana-Farber Cancer Institute Molecular Biology Core Facilities prepared libraries from 2 ng of DNA, using the ThruPLEX DNA-seq Kit (#R400427, Rubicon Genomics), according to the manufacturer’s protocol; finished libraries were quantified by the Qubit dsDNA High-Sensitivity Assay Kit (#32854, Thermo Fisher Scientific), using an Agilent TapeStation 2200 system using D1000 ScreenTape (# 5067-5582, Agilent), and by RT-qPCR using the KAPA library quantification kit (# KK4835, Kapa Biosystems), according to the manufacturers’ protocols; ChIP-seq libraries were uniquely indexed in equimolar ratios and sequenced to a target depth of 40 mol/L reads on an Illumina NextSeq 500 run, with single-end 75 bp reads. Burrows–Wheeler Aligner (version 0.6.1) was used to align the ChIP-seq datasets to build version NCB37/MM9 of the mouse genome. Alignments were performed using default parameters that preserved reads mapping uniquely to the genome without mismatches. Bam files were concatenated to sum the biological replicates of each state, and bigwiggle files were calculated for comparing the different states.

### MR-TF analysis

Enhancer and superenhancer (SE) calls were obtained using the Rank Ordering of Super Enhancer algorithm. Clique enrichment scores (CES) for each TF were calculated using clique assignments from Coltron (bioRxiv 345413). Coltron assembles transcriptional regulatory networks (cliques) based on H3K27 acetylation and TF-binding motif analysis. The CES for a given TF is the number of cliques containing the TF divided by the total number of cliques. We incorporated ATAC-seq data to restrict the motif search to regions of open chromatin. Using the CES, we performed clustering (distance = Canberra, agglomeration method = ward.D2) considering the union set TFs. One-tailed *t* test was performed to select TF-specific SKO and DKO (*P* value = 0.05). Motifs found by Coltron were represented by sequence logos (ggseqlogo R package) using the genomic region coordinates from *Mus musculus* (mm9). Integrative Genomics Viewer version 2.8.13 was used to visualize normalized ChIP-seq read counts at specific genomic loci.

### *Mybl2* CRISPR-KO cell lines

For CRISPR/Cas9-mediated KO cell line generation in DKO and PPKO cells, a two-step viral infection method was used. Guide RNA (gRNA) sequences CAT​GAC​CTG​TCA​TCC​GAC​CA and the combination of three guides TCT​GGA​TGA​GTT​ACA​CTA​CC, TTG​AAT​CCC​GAC​CTT​GTT​AA, and GAG​GTT​TCC​CAG​CCG​AGT​CC targeting murine *Mybl2* were cloned into the lenti-CRISPR puro (Addgene, #52963) according to the Zhang Lab protocol. The scrambled gRNA sequences ACA​ACT​TTA​CCG​ACC​GCG​CC and GAG​CTG​GAC​GGC​GAC​GTA​AA were used as a negative control (Ctl), respectively, in DKO and PPKO cells. The Cas9–blasticidine vectors (Addgene, 96924) were used to generate the lenti-Cas9 virus. Viral infection was performed sequentially as described by the RNAi consortium (Broad Institute) laboratory protocol “Lentivirus production of shRNA or ORF-pLX clones.” After first infection with the lenti-gRNA viruses, single clones were isolated following puromycin (2 μg/mL; Sigma-Aldrich #P8833) treatment. After second infection with the lenti-Cas9 virus, single clones were isolated following blasticidin (10 mg/mL, InvivoGen, #ant-bl) treatment.

### RNA sequencing analysis

The DKO and PPKO Ctl and *Mybl2* KO spheroids (described above) were cultured for 21 days, and RNA was extracted using the TRIzol protocol according to the manufacturer’s instructions. RNA sequencing (RNA-seq) data were aligned with STAR to mouse reference genome mm10 (GRCm38), quantified using RSEM and normalized. Gene set enrichment analysis (GSEA) was performed in GenePattern (genepattern.org). Genotypes were compared as described, using 10,000 gene set permutations to generate normalized enrichments scores, with FDR *q* value <0.25 considered significant.

### Growth in low attachment assay

For the growth in low attachment assay, 500 cells from DKO Ctl and *Mybl2* KO lines and 350 cells from PPKO Ctl and Mybl2-KO lines resuspended in 100 mL of medium were seeded in U-bottom, ultra-low attachment plates (#7007, Corning Life Sciences, Switzerland) and incubated for 5 days at 37° C under 5% CO2. ATP levels were measured using the RealTime-Glo MT Cell Viability Assay (Promega, # PRG9713) according to the manufacturer’s instructions. The luminescent signal was detected using the SpectraMax iD3 reader with a 0.5-second measuring time.

### Cell proliferation assay

DKO and PPKO Ctl and Mybl2 KO spheroids were treated with 10 µmol/L EdU for 2 hour and stained with Invitrogen Alexa Fluor 647 picolyl azide, according to the protocol for the Invitrogen Click-iT Plus EdU Alexa Fluor 647 Flow Cytometry Assay Kit (Thermo Fisher Scientific, #C10424), followed by staining with PI staining. Cells were then analyzed by flow cytometry using either 647 nm excitation (for Click-iT EdU Alexa Fluor 647 dye) or 493 nm excitation (for PI stain).

### *In vivo* therapy experiment

DKO and PPKO cells were subcutaneously implanted into syngeneic C57BL/6N mice (The Jackson Laboratory). Subsequently, 7 days after the implant, mice were treated with either 400 mg kg^−1^ of CPS2 (MedChemExpress, #HY-141680) or 2.5% DMSO in corn oil by intraperitoneal injection every 7 days. Tumors and mouse weight were measured three times weekly by caliper measurements. Treatment toxicities were assessed by body weight, decreased food consumption, signs of dehydration, hunching, ruffled fur appearance, inactivity, or nonresponsive behavior.

### IHC staining and quantification of subcutaneously injected tumors

For all stainings, 5-μm thick sections were cut from paraffin-embedded blocks and dried onto positively charged microscope slides. The following primary antibodies were used: Ki67 (Thermo Fisher Scientific, D3B5, 1:100) and phospho-H2AX (Cell Signaling Technology, 20E3, 1:100). Tissue sections were stained using the ImmPRESS HRP Anti-Rabbit IgG (Peroxidase) Polymer Detection Kit (Vector Laboratories). Tissue sections were imaged using a BZ-X810 microscope (Keyence). Images were de-identified, and five random fields per section were run through QuPath image analysis software (26) to quantify positive DAB-stained cells (as a percentage of total cells).

### Immunofluorescence staining

For CDK2 staining (2546S, Cell Signaling Technology), 5-μm thick sections were cut from paraffin-embedded blocks and dried onto positively charged microscope slides. The CDK2 primary antibody (1:100) was incubated overnight. Secondary antibody coupled to Alexa Fluor 647 (Invitrogen, A32849; 1:400) was used. Finally, the sections were stained with Invitrogen Hoechst 33342 for 5 minutes, and then the slides were mounted with ProLong mounting media (antifades; Thermo Fisher Scientific). Tissue sections were imaged using Thermo Scientific Invitrogen EVOS FL Auto 2 Imaging System (Thermo Fisher Scientific). Images were de-identified, and five random fields per section were run through QuPath image analysis software to quantify positive CDK2-stained cells.

### Western blotting

Cell lysates for Western blotting were prepared using RIPA buffer (Sigma) containing a protease and phosphatase inhibitor cocktail (Roche). Total protein (20 μg) was subjected to SDS-PAGE electrophoresis and then transferred to a nitrocellulose membrane. The membrane was blocked in 5% nonfat milk for 1 hour at room temperature, followed by primary antibody incubation [Mybl2 (MABE886, MilliporeSigma) CDK2 (2546S, Cell Signaling Technology) and CDK5 (14145S, Cell Signaling Technology)] and αGAPDH (2118S, Cell Signaling Technology). Membranes were then incubated with anti–mouse IgG (CST 7076S) or anti–rabbit IgG (CST 7074S) horseradish peroxidase–linked antibodies diluted at 1:10,000. The immunostaining was visualized using a chemiluminescent substrate kit (Thermo Fisher Scientific).

### Data availability

ChIP-seq, ATAC-seq, and RNA-seq data generated in this study are available on the Gene Expression Omnibus (GEO) at GSE271327, GSE271328, and GSE271412. The use of previously published RNA-seq data included murine prostate cancer (23, 27) and were obtained from GEO (Accession no. GSE90891 and GSE92721). Human prostate cancer gene expression data from TCGA-PRAD (408 tumor samples) were obtained from the NCI Genomic Data Commons Data Portal. The Stand Up To Cancer prostate dataset was obtained from the cBioPortal (28, 29). LUCaP gene expression data were obtained from the GEO (accession no. GSE126078). The human gene expression dataset for patients with CRPC adenocarcinoma (CPRC-Ad) and CRPC-NE (30) was obtained through dbGaP (dbGaP phs000909.v.p1).

## Results

### MYBL2 as a putative MR-TF in murine prostate cancer deficient for Rb1

**Figure 1:**
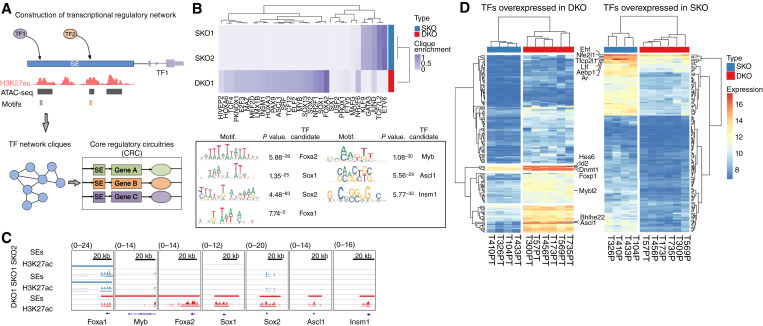
MYBL2 is a MR-TF in murine prostate cancer deficient for Rb1. **A,** Schematic representation of the Coltron algorithm procedures for the construction of transcriptional regulatory networks. **B,** Candidate MR-TFs in SKO and DKO based on regulatory CESs (range 0–1, two sided *t* test *P* value <0.05) and nucleotide motif logos for top candidate factors relative size of each base indicates the relative frequency at that position. Foxa2, Sox1, Sox2, Myb, Ascl1, and Insm1 motifs with the more significant *P* value for more abundant motifs within H3K27ac-bound chromatin were the most enriched in the DKO sample. **C,** ChIP-seq tracks of H3K27ac signal at SE regions for Foxa1, Myb, Foxa2, Sox1, Sox2, Ascl1, and Insm1 in SKO (blue) and DKO (red) samples. **D,** Heatmaps representing the expression profile of 128 TFs overexpressed in SKO samples (FDR </= 1-% log_2_ fold change >2; clustering method = “complete;” clustering distance = “Euclidean”) and 139 overexpressed DKO samples (FDR </= 1-% log_2_ fold change >2; clustering method = “complete;” clustering distance = “Euclidean”).

It has been previously demonstrated in human patients with prostate cancer that chromatin remodeling can govern tumoral phenotypes (31–33). Specifically, *de novo* expression and utilization of SEs identified by acetylation of histone H3 lysine 27 (H3K27ac) are demonstrated to enable activation of novel transcriptomes driving progression of prostate cancer (34, 35). To identify novel candidate MR-TFs, we employed a motif-based TF connectivity approach analysis using Coltron (bioRxiv 345413) from prostate cancer GEMMs. This approach scans for TF motifs inside regions of open chromatin (defined by genotype-specific ATAC-seq profiles) nestled within SE to identify TF network “cliques” (Fig. 1A). Factors are only nominated as candidates if their promoter is active (determined by H3K27ac) and if there is evidence of autoregulation defined by their cognate motif being present within the associated SE (Fig. 1A). Focusing on the comparison between GEMM-derived prostate tissue with specific deletion of *Pten* (SKO) and *Pten:Rb1* deletion (DKO), we identified candidate MR-TFs exclusive in DKO prostate samples (Fig. 1B and C; Supplementary Table S1). A total of 27 TF candidates including known NE factors *Foxa2*, *Sox2*, *Sox1*, *Insm1*, *Hes6*, and *Ascl1* showed clique enrichment in DKO, whereas *Etv6*, *Tcf7l2*, *Gata3*, and *Klf9* had higher clique enrichment in SKO tissues (Fig. 1B and C). Both SKO and DKO samples exhibited enrichment of H3K27ac at the SE region of *Foxa1* (Fig. 1C) in line with previous publications, which demonstrates that FOXA1 drives prostate cancer initiation and progression and plays an important role in prostate cancer phenotypic plasticity (36, 37).

The TFs overexpressed in SKO included *Ar*, *Ehf*, and *Nfe2l1* (Fig. 1D). Both *Ar and Ehf* are well known as important TFs in epithelial differentiation, and their downregulation promotes epithelial–mesenchymal transition and cell dedifferentiation (38, 39).

### MYBL2 expression and activity are enriched in human neuroendocrine prostate cancer and NE-like mouse models

**Figure 2:**
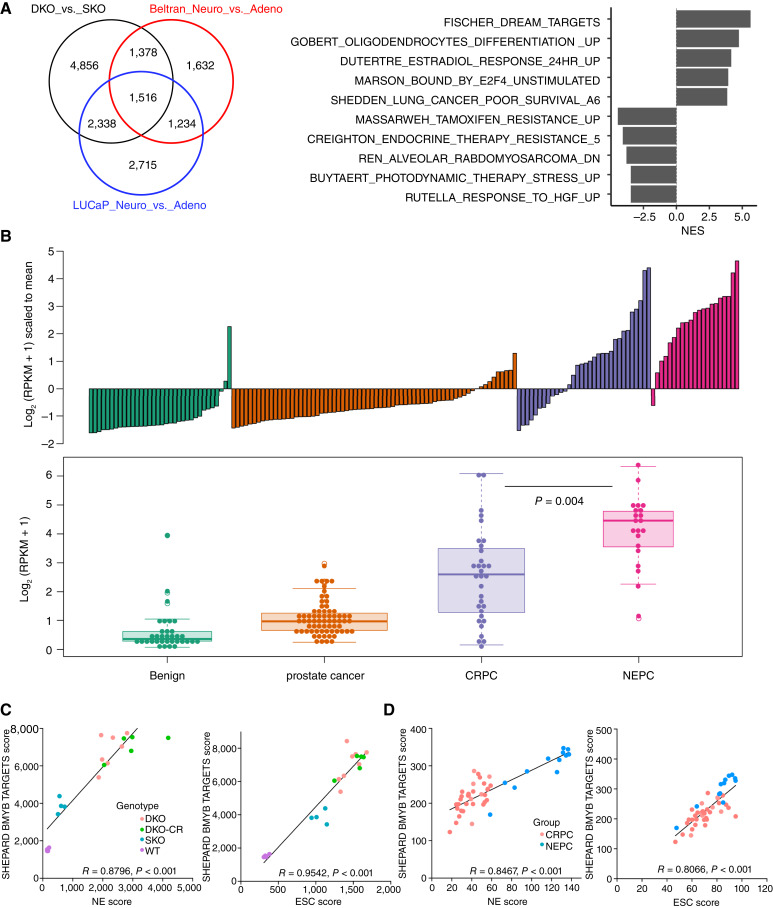
MYBL2 expression and activity is enriched in human NEPC and NE-like mouse models. **A,** Venn diagram integrating the differential gene expression (DEG) list from PBCre4:Ptenf/f (SKO) vs. PBCre4:Ptenf/f:Rb1f/f (DKO) mouse models, human NEPC vs. adenocarcinoma samples, and patient-derived xenografts of LuCaP obtained from human patients with NEPC vs. patients with adenocarcinoma shows that the three independent datasets shared 1,516 genes (left). GSEA of the 1,516 shared genes indicates that the fischer_dream_targets genes may be involved in the regulation of CRPC-AI development (right). All the pathways listed are statistically significant with *P* < 0.05. **B,** Bar and box plot summarizing the MYBL2 expression level in human patients with prostate cancer at different stages of the disease progression. **C,** Correlation analysis mouse and (**D**) human samples indicate that enrichment of MYBL2 transcriptional function is highly associated with NE and ESC gene signatures in NEPC models and patient samples compared with their adenocarcinoma counterparts. WT, wild type.

To help identify novel MR-TFs, we analyzed publicly available RNA-seq data from GEMMs (23), patient-derived xenografts (40), and human patients with prostate cancer (Fig. 2A–C; ref. 31). The integration of the differential gene expression of the three datasets [comparing DKO vs. SKO tumors, human neuroendocrine prostate cancer (NEPC) vs. adenocarcinoma, and the patient-derived xenograft samples of LuCaP NEPC vs. adenocarcinoma] generated a list of 1,516 common genes (Fig. 2A; Supplementary Table S2). GSEA indicated that the *fischer_dream_target* genes as the most enriched gene set in NEPC samples (Fig. 2A). *Mybl2* is the only member of the Myb family regulated by the DREAM complex. Looking over the spectrum of disease progression in human prostate cancer samples, tumors from patients with NEPC represented the most enriched sample set for increased *MYBL2* expression. Of interest, a subset of samples from patient with CRPC (adenocarcinoma) did exhibit medium-to-high levels of *MYBL2* (Fig. 2B); however, overall *MYBL2* expression was significantly increased in NEPC samples. Moreover, we observed that *MYBL2* transcriptional activity was most positively correlated in murine (Fig. 2C) and human (Fig. 2D) with NE and embryonic stem cell (ESC) gene signatures in NEPC samples versus adenocarcinoma. Although increased MYBL2 expression in prostate cancer is linked to resistance to androgen receptor signaling inhibitors (ARSI), it remains unknown if MYBL2 mechanistically increases the potential for prostate cancer to activate alternate lineages within the phenotypic plasticity spectrum when progressing on AR-targeted treatments. Furthermore, whether MYBL2 stands as a biomarker of disease progression and can indicate therapy options are questions currently not answered.

### Mybl2 supports stemness and cell fitness in prostate cancer models

**Figure 3:**
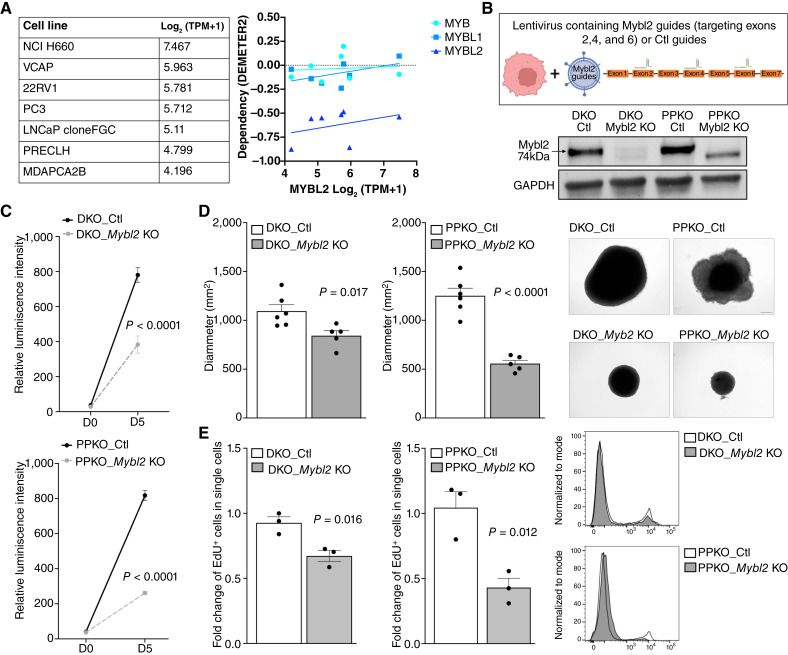
Mybl2 supports tumoral proliferation and promotes self-renewal in prostate cancer cell line models. **A,** Normalized gene expression of *MYBL2* in human prostate cancer cell lines. DEMETER gene dependency of MYB family members across human prostate cancer cell lines indicating *MYBL2* as a significant dependency compared with other family members. **B,** Schematic representation of the DKO Ctl and DKO *Mybl2* KO and PPKO Ctl and PPKO *Mybl2* KO cell line generation using the two-step CRISPR/Cas9 method. Western blot indicating Mybl2 protein levels in the DKO Ctl, DKO *Mybl2* KO, PPKO Ctl, and PPKO *Mybl2* KO generated cell. **C,** Growth kinetics representation using relative luminescence intensity of DKO Clt vs. DKO *Mybl2* KO spheroids and PPKO Ctl vs. PPKO *Mybl2* KO spheroids cultured from day 0 (D0) to day 5 (D5) in a low-attachment round-bottom 96-well plate. **D,** Spheroid diameter and pictures of the DKO vs. DKO *Mybl2* KO and PPKO vs. PPKO *Mybl2* KO cells for 21 days postseeding of 500 or 350 cells/well, respectively. **E,** Flow cytometry analysis of the EdU-positive cells in DKO vs. DKO *Mybl2* KO and PPKO vs. PPKO *Mybl2* KO cells after 2 hours of EdU labeling *in vitro*.

Given the role of MYBL2 in proliferation (41), genome stability (42–44), and reprograming (17), we sought to understand cellular consequences when *Mybl2* expression was lost in murine prostate cancer cell lines. First, we used the DepMap database (45) through the Broad Institute to determine the dependency of the MYB genes in human prostate cancer cell lines. In line with our patient data, the human NE cell line H660 exhibited the highest expression of all MYB genes. Interestingly, MYBL2 was the only family member that represented a genetic dependency in all cell lines (Fig. 3A). We validated genetic dependency by using independent murine prostate cancer cell lines generated from GEMMs representing phenotypic plasticity. We observed that CRISPR/Cas9 deletion of *Mybl2* (Fig. 3B; Supplementary Fig. S1A) did not disrupt the overall growth potential of these cells under anchorage-dependent 2D culture conditions (Supplementary Fig. S1B). However, under anchorage-independent three-dimensional culture conditions through use of a growth in low attachment assay, we observed a significant loss in spheroid formation ability. In addition, loss of Mybl2 also repressed cell growth associated with significant loss in DNA replication (Fig. 3C–E).

**Figure 4:**
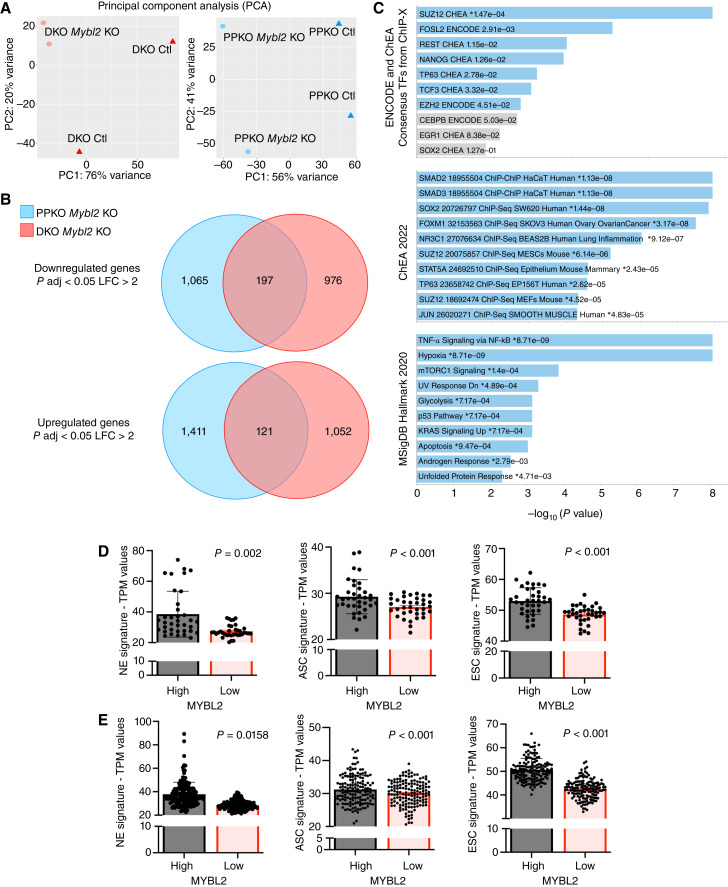
Increased Mybl2 expression supports stem-related gene signatures in murine and human prostate cancer. **A,** Two-dimensional principal component analysis visualization of bulk RNA-seq analysis performed in DKO Ctl vs. DKO *Mybl2* KO cells (left) and PPKO Ctl vs. PPKO *Mybl2* KO cells. **B,** Venn diagram representation of down- and upregulated genes from the DEG analysis between DKO *Mybl2* KO vs. DKO Ctl cells and PPKO *Mybl2* KO vs. PPKO Ctl cells showed that both cell lines shared 197 down and 127 upregulated genes. The *P* adjusted value used to calculate the DEG was <0.05 and the LFC was < or > 2. **C,** Bar chart visualization of the top 10 enriched terms and their *P* values using ENCODE and ChIP-x enrichment analysis (ChEA), ChEA 2022, and Hallmarks 2020 databases using the 197 downregulated genes shared between DKO *Mybl2* KO and PPKO *Mybl2* KO cells. All pathways listed were statistically significant with *P* < 0.05. **D,** Normalized gene expression values for TCGA-PRAD and (**E**) SU2C samples from patient with CRPC show high *MYBL2* expression (top 25% of samples) is associated with significantly increased expression of gene signatures for NEPC (NE), ASC, and ESC signatures when compared with patient samples with lowest *MYBL2* expression (bottom 25% of samples).

Gene expression analysis by RNA-seq revealed that the loss of *Mybl2* expression induced a dramatic change in overall transcriptome profiles (Fig. 4A). Furthermore, comparing the differential gene expression between *Mybl2* KO with their respective Ctls, we found 197 shared downregulated and 121 upregulated genes between both cell lines (Fig. 4B; Supplementary Table S3). In line with our observations in Figs. 2 and 3, GSEA demonstrated a significant repression of gene sets associated with pluripotency, stemness, and reprogramming including *Fosl2*, *Ezh2*, *Suz12*, *Rest*, *Sox2*, *Foxm1*, *Tp63*, and *Nanog* (Fig. 4C; refs. 46–48). Based on initial observations from Fig. 2B, in which a subset of CRPC-Ad samples displayed increased gene expression for *MYBL2*, we extended this analysis to samples from patients with adenocarcinoma in TCGA (Fig. 4D) and SU2C (Fig. 4E) datasets. Comparing the *MYBL2* expressing top and bottom 25% of samples, we observed that high *MYBL2* expression was associated with increased NE, adult, and ESC gene signatures. These data indicate that MYBL2 promotes pluripotency and stemness networks associated with phenotypic plasticity and can stand as a genetic dependency in prostate cancer models.

### Mybl2 transcriptional activity identifies CDK2 as a novel therapy target for phenotypic plastic prostate cancer

**Figure 5:**
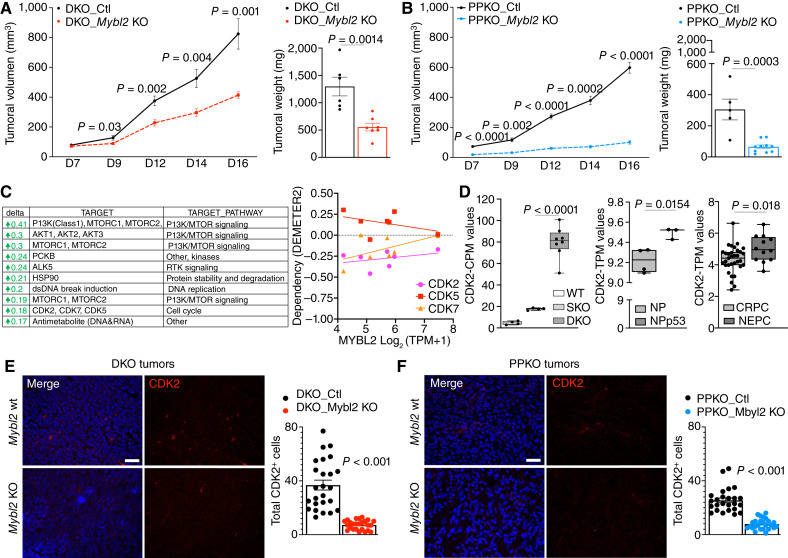
Mybl2 is a genetic dependency associated with CDK2 expression in NEPC. **A,** Tumoral growth curve of murine DKO Ctl and DKO *Mybl2* KO cells and (**B**) PPKO Ctl cells and PPKO Mybl2 KO cells in NOD SCID mice. Tumor growth was monitored by serial caliper measurements every second day for 16 days (D16). Tumor weight was measured at D16 after dissection (*n* = 5 mice per treatment group (±1 SEM). **C,** Top responses from the cancer therapeutics response portal analysis using a MYBL2 gene signature. DEMETER gene dependency of CDK family members 2, 5, and 7 across human prostate cancer cell lines indicating as significant, as outlined in Fig. 3A. **D,** Box plot graphs summarizing the RNA-seq analysis from published prostate cancer GEMMs (first and second graphs, including wild-type, SKO, DKO, NP:*Nkx3.1*^CreERT2^:Ptenf/f and NPp53:*Nkx3.1*^CreERT2^:Ptenf/f:Trp53f/f), and human samples (third graph, including CRPC and NEPC samples) indicate CDK2 upregulation in NEPC mouse models and human patients. **E,** Immunofluorescent IHC staining for CDK2 in DKO and (**F**) PPKO murine tumors indicates a significant loss of CDK2 expression following MYBL2 KO.

To evaluate the contribution of *Mybl2* expression in tumoral growth *in vivo*, mice were injected subcutaneously with DKO or PPKO cells with *Mybl2* wild-type (Ctl) or *Mybl2* KO cells. Loss of Mybl2 expression significantly reduced the potential for overall *in vivo* tumor formation and growth (Fig. 5A and B). These data highlight MYBL2 as a promising therapeutic target, but currently no direct targeted therapies toward MYBL2 are known. Therefore, we used an established MYBL2 gene signature (26) to identify and nominate candidate drug targets as a novel therapeutic direction using the cancer therapeutics response portal for patients with prostate cancer with overexpression of MYBL2 and/or increased MYBL2 activity (Fig. 5C). From this analysis, numerous candidate druggable pathways were nominated including PI3K/mTOR signaling, RTK signaling, protein stability and degradation, and DNA replication. Of most interest to us, due to potential novelty for treating phenotypic plastic prostate cancer, were the cell cycle–related CDKs, which included CDK2, CDK5, and CDK7. Based on information gained from this analysis and the success of CDK inhibitors in other cancers (49, 50), we applied the DEMETER computational method (45) to infer the dependency of these three CDK genes in the human prostate cancer cell lines mentioned in Fig. 3. We found that CDK2 demonstrated consistent dependency within all prostate cell lines including the H660 cell line (Fig. 5C). In line with these dependency data, we observed that NE-like GEMMs and patients with NEPC had significantly higher *cdk2* expression when compared with their adenocarcinoma counterparts (Fig. 5D). CDK2 is a transcriptional target of MYBL2, which holds true in both NE-like murine tumor models, in which a significant reduction in CDK2 expression occurred in tumors with MYBL2 KO (Fig. 5E and F).

**Figure 6:**
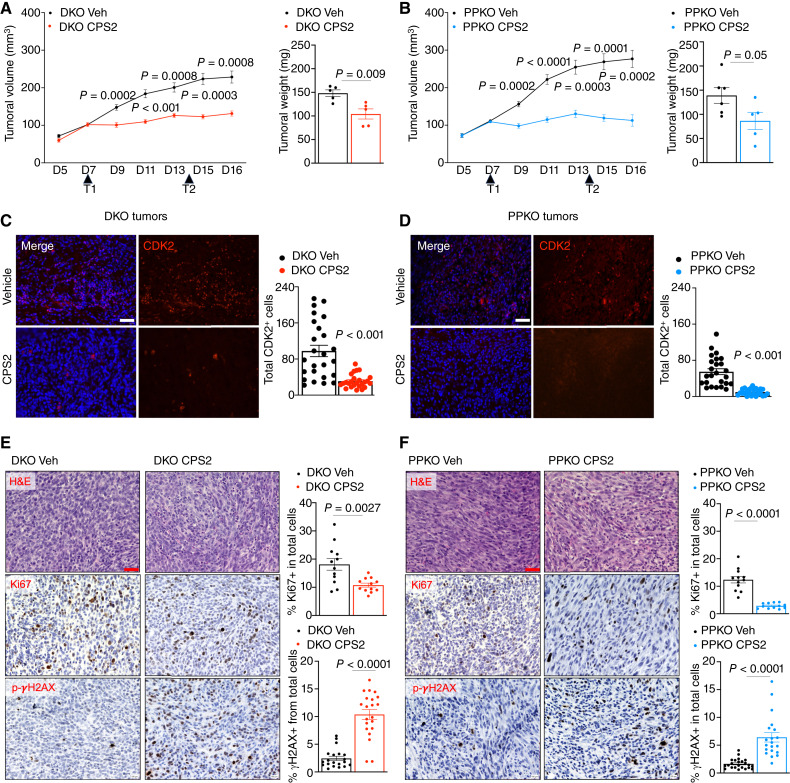
Inhibition of CDK2 represents a novel therapeutic target for NEPC. **A,** Tumoral growth curve of murine DKO cells in C57BL/6N and (**B**) PPKO cells in C57BL/6N mice and treated with either vehicle control or the CDK2 PROTAC—CPS2. Mice were treated every 7 days (D7 and D14) with CPS2 (400 mg/kg) by intraperitoneal injection. Tumor growth was monitored every second day for 16 days (D16) by serial caliper measurements. Tumoral weight was measured at D16 after dissection (*n* = 5 mice per treatment group (±1 SEM). **C,** Immunofluorescent IHC staining for CDK2 in DKO and (**D**) PPKO tumors show that CPS2 inhibition significantly reduces CDK2 expression. **E** and **F,** Example pictures of hematoxylin and eosin, Ki67, and p-γH2AX IHC staining in murine DKO and PPKO tumors from the *in vivo* study and corresponding quantification of the percentage of positive nuclei from total cells (*n* = 5 mice per treatment group, ±1 SEM).

To investigate the potential targeting of CDK2 in phenotypic plastic tumors, we initially tested a CDK2 inhibitor (BGG463; refs 51, 52) *in vitro* (Supplementary Fig. S2A). Response to BGG463 was more significant in DKO and PKO murine cell lines when compared SKO cell response. Although BGG463 demonstrated superior efficacy in phenotypic plastic prostate cancer cell lines, it is shown that BGG463 can exhibit indirect inhibition of alternate kinase function not related to CDK2, particularly inhibition of T315I BCL-ABL autophosphorylation in murine models of chronic myeloid leukemia (ref. 52). For this reason and wanting to test a more efficient on-target inhibitor of CDK2, we utilized the CDK2 degrader CPS2 (53). Because of initial superiority of response, we only tested CPS2 ability to directly degrade CDK2 *in vitro* using DKO and PPKO cells. Previous analysis (53) showed that CPS2 did specifically degrade CDK2, and we repeated these results in DKO and PPKO cells, showing direct downregulation of CDK2 expression when compared with CDK5, which remained stably expressed (Supplementary Fig. S2B–S2E). *In vivo* treatment of C57BL/6N wild-type mice harboring DKO or PPKO tumors confirmed the ability of CPS2 treatment to significantly reduce the overall growth of DKO and PPKO tumors (Fig. 6A and B), which phenocopied the loss of Mybl2 expression (Fig. 5A and B). In addition, CPS2 treatment significantly depleted CDK2 expression in both DKO and PPKO tumors (Fig. 6C and D). Moreover, CPS2 treatment response in both DKO and PPKO tumors was confirmed by using IHC to show that loss of tumor growth was associated with loss of overall proliferation as indicated by lower Ki67 nuclear staining and increased DNA damage indicated by elevated nuclear staining for γH2AX (Fig. 6E and F).

## Discussion

Despite the advancement of available therapy options for patients with prostate cancer with mCRPC, progression is still inevitable. Although most tumors progress and maintain AR dependency, approximately 15% to 20% patients will progress where their tumor is indifferent to AR signaling (CRPC-AI) and activate developmental programs related to stemness and pluripotency (phenotypic plasticity). For these patients, treatment options remain extremely limited, and it is imperative that novel therapeutic approaches are identified.

In this study, we identified MYBL2 as a putative MR-TF driving progression to CRPC-AI. These results were in agreement with previous publications in which MYBL2 was characterized as a putative oncogene (54), promoting the malignant progression of tumors by controlling cancer cell proliferation, therapy resistance, and metastasis (21). Although MYBL2 is implicated in CRPC-Ad growth (55, 56), no studies have examined the specific functional and clinical implications of increased MYBL2 expression in driving phenotypic plasticity in prostate cancer. MYBL2 expression and function were increased as localized prostate cancer progresses to CRPC-Ad (19, 57). However, our work demonstrates that MYBL2 is more significantly increased in mouse models representing phenotypic plasticity and patients with NEPC.

Given the role of MYBL2 in proliferation (41), we asked whether the loss of *Mybl2* impaired the growth of NE-like murine prostate cancer cells. Surprisingly, loss of *Mybl2* in DKO cells did not inhibit the overall growth of 2D cell cultures. However, using a three-dimensional sphere-formation assay, loss of *Mybl2* did significantly perturb sphere-forming efficiency involving loss of DNA replication and indicating a dependence for *Mybl2* expression in maintaining a higher self-renewal capacity. Gene expression analysis further supported this by indicating that *Mybl2* mediated transcriptional programs related to self-renewal and pluripotency, as well as increased stem cell signatures in samples from patients with NEPC and patients with adenocarcinoma with highest MYBL2 expression. Our data are in line with others, in which overexpression of MYBL2 is shown to inhibit neural and glial differentiation induced by retinoic acid (58). Additionally, MYBL2 is known to be critical for the development of hematopoietic stem cells (59) and coordinates self-renewal and pluripotency by regulating expression of pluripotency genes including *POU5F1*, *SOX2*, and *NANOG* in ESCs (15, 60, 61).

Although the MYB family of TFs, including MYBL2, is emerging therapeutic targets in hematologic and solid malignancies, no direct MYB targeting therapy has been identified. Using a MYBL2 gene signature (26), we identified CDK2 as a candidate therapeutic target. MYBL2 and CDK2 are molecularly linked as CDK2 is a transcriptional target of MYBL2 (62), and CDK2 phosphorylates MYBL2 to enhance transactivation (63). Indeed, we demonstrate that CDK2 inhibition presents a more robust antitumor response *in vivo* using our phenotypic prostate cancer tumor models. Both CDK2 (64) and MYBL2 (65, 66) are implicated in DNA damage repair response; however, more insight is needed to determine if there are co-dependencies requiring cooperation between CDK2 and MYBL2 for this purpose.

Together, our study provides evidence that MYBL2 is an important MR-TF driving stemness in NEPC, and this increase in transcriptional function of MYBL2 highlights that NEPC would be susceptible to inhibition of CDK2. Previously, MYBL2 mRNA expression has been suggested as a biomarker to identify patients with myelodysplastic syndrome that would respond to genotoxic treatments (67). These data raise rationale that CDK2 inhibition should be candidate target taken to clinical trial to test toward the treatment of patients with NEPC.

## Supplementary Material

Supplement Figure 1 A. Full membrane following CRISPR targeting of Mybl2 indicating specific targeting of our target gene. B. DKO-Ctl and DKO-Mybl2 KO cells were cultured as 2-dimensional monolayers and overall cell growth was determined by CellTiter-Glo luminescent cell viability assay.

Supplement Figure 2 Supplement Figure 2. A. SKO, DKO, and PPKO were cultured as 3-dimensional spheroids and treated with the indicated concentrations of the CDK2 inhibitor - BGG463 over 72 hours. Cell growth was determined by CellTiter-Glo luminescent cell viability assay. B. Western blot analysis for Cdk2, Cdk5, and Vinculin following DKO and PPKO were treated with the indicated concentration of the CDK2 PROTAC - CPS2. Full western blot membranes for C) CDK2, D) CDK5, and E) vinculin.

Table S1 Summary of Peak calls following H3K27ac ChIPseq with Atacseq integration.

Table S2 Combined genes enriched in human NEPC, PDX samples and NE-like mouse models

Table S3 DEG analysis between DKO Mybl2 KO versus DKO Ctl cells and PPKO Mybl2 KO versus PPKO Ctl
