# Supplementary figures and images for: MYBL2 Drives Prostate Cancer Plasticity: Inhibiting Its Transcriptional Target CDK2 for RB1-Deficient Neuroendocrine Prostate Cancer
Source: Cancer Res Commun. 2024 Sep 2;4(9):2295–307. doi: 10.1158/2767-9764.CRC-24-0069 (PMC11368174; doi:10.1158/2767-9764.CRC-24-0069)

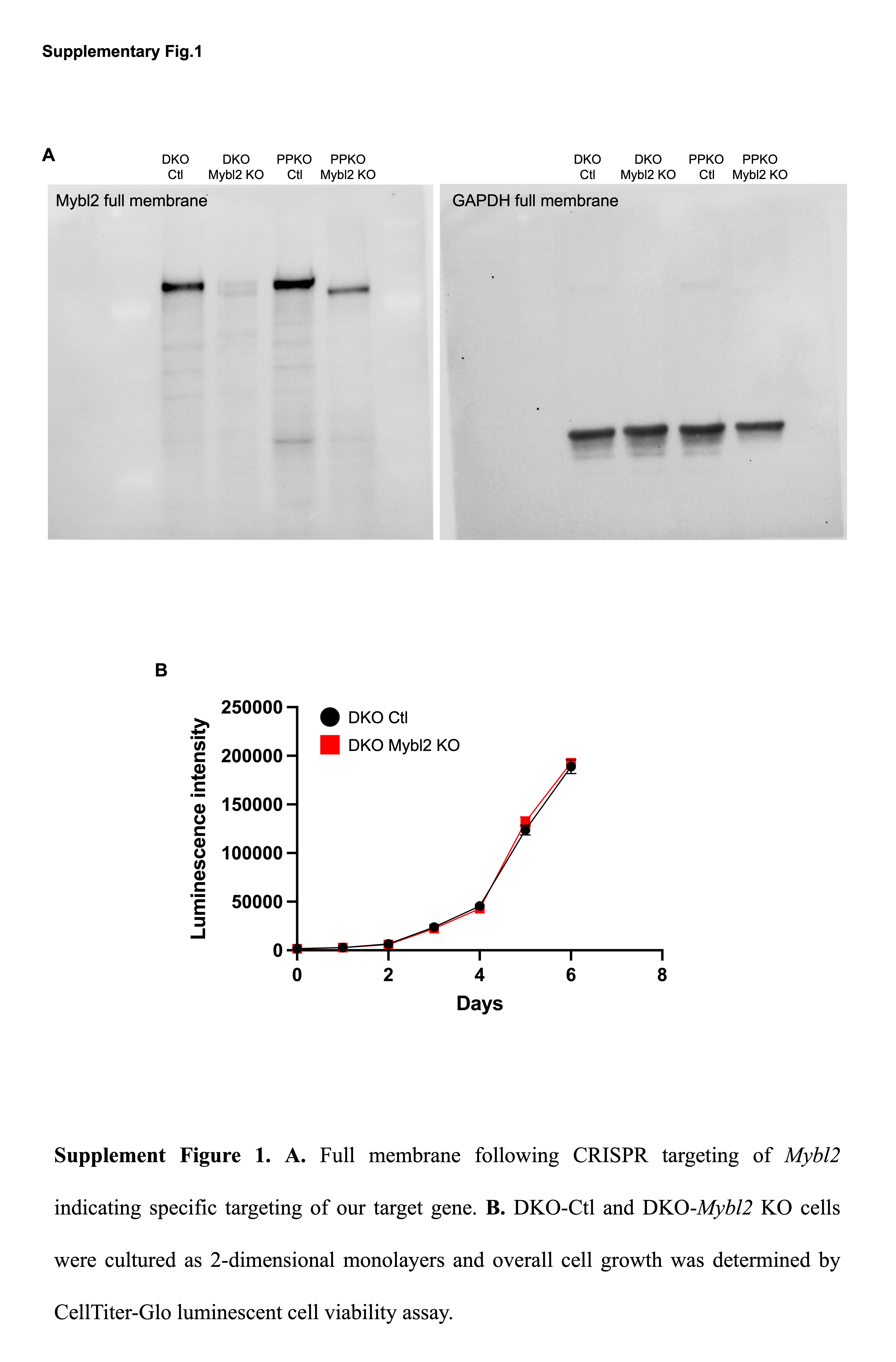

Supplement: Supplement Figure 1 — A. Full membrane following CRISPR targeting of Mybl2 indicating specific targeting of our target gene. B. DKO-Ctl and DKO-Mybl2 KO cells were cultured as 2-dimensional monolayers and overall cell growth was determined by CellTiter-Glo luminescent cell viability assay. [file crc-24-0069_supplement_figure_1_suppsf1.png]

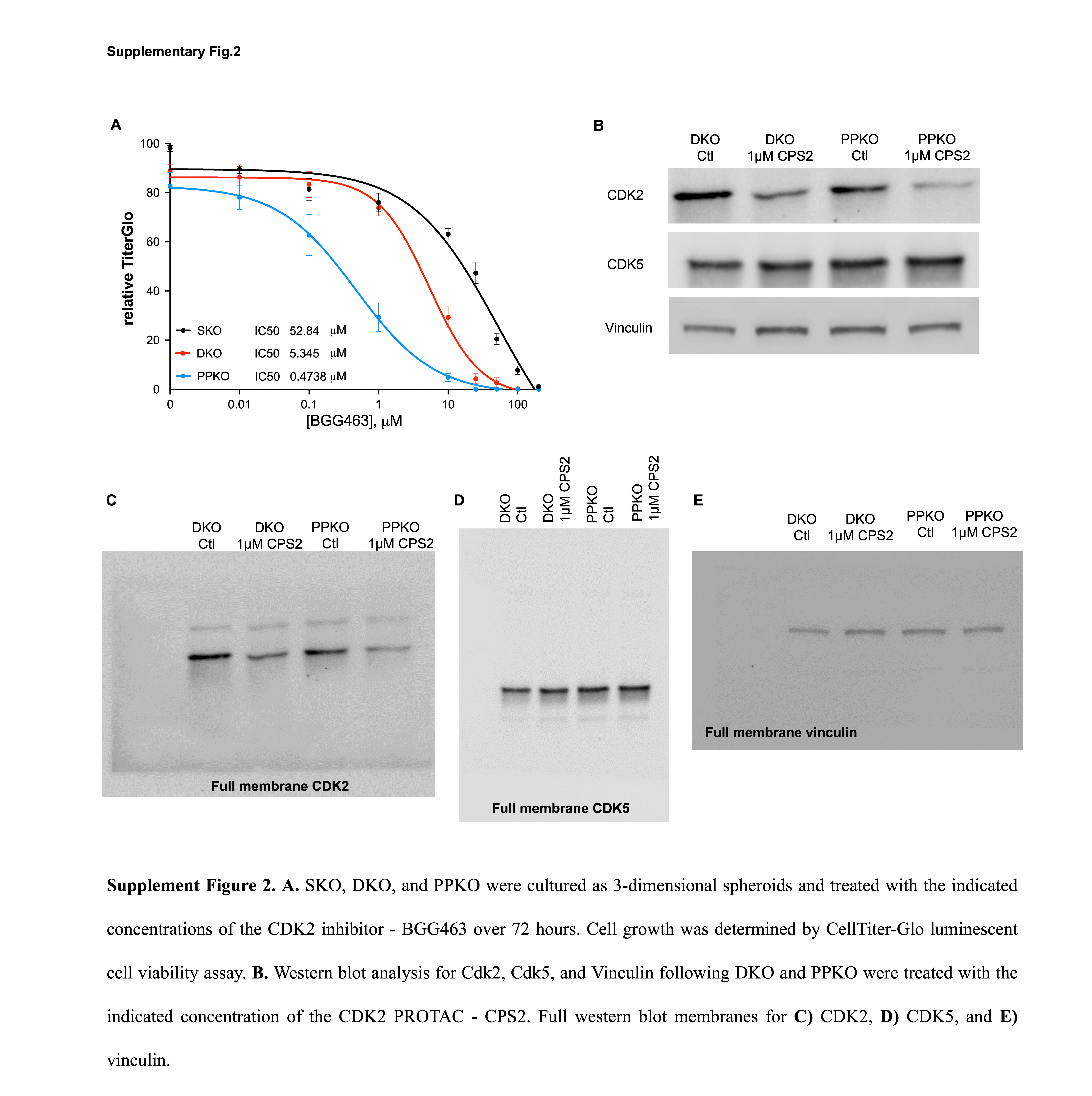

Supplement: Supplement Figure 2 — A. SKO, DKO, and PPKO were cultured as 3-dimensional spheroids and treated with the indicated concentrations of the CDK2 inhibitor - BGG463 over 72 hours. Cell growth was determined by CellTiter-Glo luminescent cell viability assay. B. Western blot analysis for Cdk2, Cdk5, and Vinculin following DKO and PPKO were treated with the indicated concentration of the CDK2 PROTAC - CPS2. Full western blot membranes for C) CDK2, D) CDK5, and E) vinculin. [file crc-24-0069_supplement_figure_2_suppsf2.png]
